# Which Seat at the Table? The Ways That Senior Service Organizations Are Engaged in Age-Friendly Community Efforts

**DOI:** 10.1093/geroni/igaa057.076

**Published:** 2020-12-16

**Authors:** Caitlin Coyle, Shayna Gleason

**Affiliations:** University of Massachusetts Boston, Boston, Massachusetts, United States

## Abstract

Senior centers across the nation continue to serve as important access and focal points for older adults to voice their desires, get basic needs met, and to engage in opportunities that support many of the key concepts of Age-Friendliness (i.e. social participation, respect and social inclusion, civic engagement, transportation, and community supports and health services). Senior centers are the front line of aging services and thus in a position to implement programs and raise public awareness about age-friendly initiatives. The purpose of this presentation is to present and discuss the involvement of senior centers or other senior service agencies, as well as to characterize the mobilization of a community after joining the movement. We will present 5 case studies of age-friendly communities who are in the implementation phase of their initiative, to illustrate the challenges, opportunities, and outcomes associated with placing a senior center at the forefront of the movement. Based on the results of this work, we will present a typology of age-friendly community initiatives as a mechanism for supporting other communities make this transition. We conclude with a discussion of how age-friendly communities are part of the paradigm shift of aging in community and the ways in which this work intersects with other health policy initiatives with which cities and towns engage.

